# Supplementary material for: Methodologies for Monitoring the Digital Marketing of Foods and Beverages Aimed at Infants, Children, and Adolescents (ICA): A Scoping Review
Source: Int J Environ Res Public Health. 2022 Jul 23;19(15):8951. doi: 10.3390/ijerph19158951 (PMC9330739; doi:10.3390/ijerph19158951)
Supplement: Supplementary file 1 [file ijerph-19-08951-s001.zip › ijerph-1782552-supplementary.pdf]

Supplementary Material 1. Included sources in the scoping review (*n* =28)

Author	Year	Type of study	Country	Objective	Advertising target population	Methodology applied	Studied digital and social media	Products of intervention	Key findings
**Breast-milk substitutes (BMS)**									
Ching, et al.	2021	Cross-sectional (mixed methods)	Burkina Faso, Canada, China, India, Indonesia, Kenya, Laos, Malaysia, Myanmar, Pakistan, Singapore, Philippines, United States, Vietnam	To examine the marketing tactics of BMS companies since the start of the COVID-19 pandemic.	Parents and families with children	For quantitative analysis, data was collected through an official database of reported violations of Executive Order 51 in the Philippines from January 2019 to July 2020. Using the database, descriptive information about violations reported by date and type was extracted. On the other hand, for qualitative analysis, promotional activities, and materials by BMS companies dating from 30 January 2020, were identified as appropriate data for this study. Purposive sampling was used to collect data via the internet, print magazines, networks of health officials and professionals, health facilities, and shops from August to October 2020.	Facebook, Instagram, and company website	Infant, follow-up, and growth formula	Broad themes emerged from the selected data after thematic analysis including unfounded health claims on immunity that prompt fear; association with public health authorities to gain legitimacy; prominent use of digital platforms to reach out to parents; promoting uncertainty through endorsing breastfeeding; discounts on BMS products that are linked to COVID-19. Regarding quantitative data 80% were related to donations of BMS and the rest was related to sponsorship, gift and mass media promotion.
Han, Shanon	2020	Thesis	China	To analyze the BMS retail websites on the largest business to consumer (B2C) e-commerce platform TMall, to characterize the marketing themes and strategies used to target consumers.	Parents and families with infants	Two aspects of each website were identified and sampled. First, the main landing page (MLP) of each flagship website was captured. The product description page (PDP) of all unique individual formula products (stages 1 to 3) listed in the comprehensive catalog of each e-commerce site were identified. The quantitative approach consisted of describing the frequency of different outcomes including thematic appeals, deviations from the Code articles, and images used. For qualitative data, texts and language used were extracted to provide the context of each variable and to examine in-depth the marketing appeals and strategies to target consumers.	e-commerce platform TMall	BMS	Descriptive results revealed that Premiumization and Science & Nutrition were the most used marketing appeal strategies among both MLP and PDP of companies' websites.<br>The marketing strategies had low adherence to the Code and national Code of Conduct, only 34.5% included pro-breastfeeding statements.<br>A total of 27.4% of the PDP samples used images of infants (<12 months), and 33.6% of PDP samples made favorable comparisons of BMS to breastmilk.

Lozada-Tequeanes, et al.	2020	Cross-sectional	Mexico	To examine the advertising and marketing of BMS through the internet, social media and television in Mexico.	Parents and families with children	Social media sites of the BMS companies operating on the Mexican market were identified by Google search. Content codification was conducted by two nutritionists. Information was coded and saved in electronic format for further analysis based on the Code's stipulations on the labeling of BMS products.	Facebook, Twitter and YouTube	Infant, follow-up and growth formula, and complementary food	Six brands available on the Mexican market were identified: Abbott, Bayer Shering Pharma Mead Johnson, Nestlé, PiSA Farmacéutica and Wyeth.<br>The presence of advertisements of BMS was observed in the three types of monitored mass media: internet, TV and social networks.<br>A total of 33 commercials for BMS were broadcasted in 1 of the 4 channels where the majority (75.7%) were for PiSA Farmacéutica products.<br>Three of the six companies had more than one website promoting their different brands.<br>The monitored Facebook accounts had an average of 0.9 posts per day about advertising a product or providing tips for feeding young children.
Pereira-Kotze, et al.	2020	Cross-sectional	South Africa	To provide pertinent examples of how BMS manufacturers in South Africa use social media to market their products thus violating national regulations.	Parents and families with children	A digital ethnography approach was used. This involved studying organizations' activity and behavior on social media platforms. The first author observed sponsored posts by BMS manufacturers on their own personal Facebook newsfeed and then actively sought out different Facebook pages and Instagram handles managed by manufacturers of BMS in SA.	Facebook and Instagram	BMS and food for infants and young children	An example of a violation of South Africa's Regulation R991 is provided from a BMS manufacturer's Facebook page from Nestlé Baby & Me' South African page where rewards were offered alongside a video that includes the Nestlé LactoKid 4 product.<br>The second example comes from PURITY's South African Instagram where cultural values like heritage were used for commercial purposes.

Prado and Rinaldi	2020	Cross-sectional	Brazil	To verify the compliance with Law No. 11,265/2006 in the promotion strategies for IF in Brazilian websites of manufacturers and drugstore networks.	Parents and families with infants	Nine drugstore networks were selected based on the survey of the largest companies in the country conducted by the Brazilian Retail and Consumption Society (2017) and for having an online store. After this selection, the term 'infant formula' was entered into the search box, and the predefined items that appeared were examined. Five infant formula manufacturers were chosen based on their existence on all the websites of the selected drugstore networks During one-month the promotion of infant formulas was examined and, the main attributes evaluated were: the use of drawings or representations of children, the presence of pop-up windows with other infant formulas or links to children's product websites, among others. Data were obtained on the total number of commercial IFs available on the websites of manufacturers and drugstores, as well as on the attributes that were inadequate/non-compliant in the websites.	Formula manufacturers' websites and drugstore networks	Infant formula	Among the main findings, at least one noncompliance with the legislation on the websites of infant formula manufacturers was identified. In addition, 66.6% of the drugstores examined did not conform to the requirement of displaying a mandatory warning statement on the feeding of babies aged up to six months and 100% did not display warnings on the feeding of babies aged six months and older.
Senkal and Yildiz	2019	Cross-sectional	European country	To examine the presence of infant formula marketing on social media.	Parents and families with infants	Qualitative research examined the presence of infant formula brands on popular social media platforms (Facebook, Twitter, Instagram, blogs, mobile applications, and interactive websites) promoting BMS (from December 2016 to January 2018). Marketing activities were examined using the World Health Organization (WHO) Code as a basis.	Facebook, Twitter, Instagram, blogs, websites, and mobile apps	Infant formula	A total of 46 accounts from nine infant formula brands were identified. Most violations were made through idealizing the use of BMS with health or nutrition claims.
IBFAN Asia & IBFAN-ICDC	2018	Report	Bhutan, China, India, Indonesia, Republic of Korea, Maldives, Mongolia, Nepal, Philippines, Sri Lanka, and Thailand	To identify the presence of violations and their predominant trends.	Parents and families with infants	Detailed monitoring process was provided in the IBFAN-ICDC Code monitoring toolkit for IBFAN-Asia regional monitoring. Each country monitor was requested to read the document carefully to add to the general understanding of monitoring. Country monitors identified all the manufacturers marketing in their respective countries. Minimum of 4 labels from each manufacturer were monitored. Code violations thus identified were sent to ICDC using an online Quick and Easy Code monitoring form provided in the Code monitoring toolkit. With the available information from countries, a regional report of the Code violations on Labeling and Online promotion of baby foods and feeding bottles has been developed.	e-marketing portals (Amazon, eBay, among others)	BMS	Promotions identified in digital media:<br>-China: Multiple shopping incentives all for Women's Day on Tmall.<br>-India: cashback offers; -Indonesia: free iPhone giveaway.<br>-Mongolia: Free shipping as promotional rewards to shoppers.<br>-Philippines: Full blown advertising on shopping website storefront.<br>Republic of Korea: Appealing to shoppers' "mother instinct"; -Sri

									Lanka: Discounts offered at online stores.<br>-Thailand: Discounts offered at online stores.
Berry and Gribble	2017	Cross-sectional	Australia	To determine whether such prohibited claims could be observed in Australian websites that advertise IF products.	Parents and families with infants	In order to identify the websites, a one-day search (July 14, 2014) was conducted using the Google search engine to approximate consumer behavior. To avoid obtaining results influenced by the researcher's previous behavior, the browser cache was cleared, and the researcher did not log in before performing the search. The search was limited to Australian sites using 'infant' and 'formula' as search terms, and then the first 10 pages related with BMS for children aged less than 12 months were selected and examined. All pages required visitors to indicate their agreement to directly view pages advertising IF products. The webpages that advertised an IF product or a brand associated with an IF were captured in printed format (using the 'print this page' function) or in a screenshot. A thematic coding frame based on the Australian and New Zealand Food Standards Code (F2016C00161) was used to identify nutrient content claims, health claims, and references to the nutrient content of human milk.	Websites advertising infant formula products	Infant formula	Twenty-five advertising websites were identified for seven brands of infant formula. The majority web pages advertising infant formula products contained more than one prohibited claim. All advertising pages identified contained at least one health claim. 72% advertising pages also contained at least one nutrition content claim.
Vinje, et al.	2017	Cross-sectional	Cambodia, Indonesia, Myanmar, Thailand, and Vietnam	To review regulations and to perform a media audit of promotion of products under the scope of the Code in South-East Asia.	Parents and families with infants	Independent media agencies were hired to conduct systematic media monitoring. The media explorations lasted three months in Vietnam and six months in the rest of the countries. Media monitoring included advertisements in print, online or television. Keywords used in the research were: breastfeeding, infant formula, follow-on or toddler milk, growing-up milk, bottles and teats, and milk for pregnant and breastfeeding women, as well as brand names of popular products. Companies and brands identified through traditional media monitoring in Cambodia, Myanmar and Vietnam were chosen, and the last 30 posts from their Facebook pages were collected by the researchers. Texts, images, and audiovisuals were examined to identify key messages, stakeholders involved, and the	Editorial content, Facebook, and Television	BMS	On mass media, 387 advertisements were collected. In Cambodia and Vietnam television was the main channel. About 80% of the advertisements were from growth formulas. On Facebook, online forums created by the BMS companies were identified in which mothers could share experiences or seek advice about infant and young child feeding. Regarding violations, advertisements of growth formulas were identified that violated article 5.1 of the Code. In addition, there was identified the use of baby pictures and

						products promoted. In addition, to estimate the trend of market size of the IF industry (from 2000 to 2014), data about annual growth in Indonesia, Thailand and Vietnam were purchased from Euromonitor International (http://www.euromonitor.com). Similar data for Cambodia and Myanmar were not available. Also, the total annual estimate of ads was calculated by identifying the number of ads and multiplying it by four in Vietnam, and by two for the other four countries (assuming that ads are distributed equally per month in each country).			feeding bottles. Regarding the market size BMS companies, was identified a value growth of market size between 2009 and 2014 was 125% in Vietnam, 96% in Indonesia and 33% in Thailand.
Gunter, et al.	2013	Cross-sectional	United Kingdom (UK)	To examine formula manufacturers' web sites to ascertain whether these are used as alternative forms of advertising that fall outside current regulations.	Parents and families with infants	From February to March of 2009, and again in March of 2012, the websites of the top five UK infant formula manufacturers were visited to identify what kind of online information was provided on infant and follow-on formulas. It should be noted that this was not a formal content analysis of the sites, however, two of the authors independently checked these sites to ensure the reliability of the coding. The mentioned authors had the skills to identify whether there was digital content that would be classified as promotional in nature (such as images of product packaging); whether it was openly stated that breastfeeding is best; and whether it was made explicit that such products should only be used under professional advice (specifically in the case of infant formula).	Formula manufacturers' websites	Infant and follow-on formulas	Some of the results identified on manufacturers' websites are described below. Formula manufacturer 1: on the website, in 2009 there were links that visitors could use to reach product information about infant and follow-on formula products. In 2012 were an advice that breastfeeding is best, and visitors could gain access to no product information about infant formula products. Formula manufacturer 2: main page website contained a prominent statement about changes to different formula milks to ensure they complied with new EC and UK regulations (2009). By 2012, availability of infant formula on the website was visible and accessible. Formula manufacturer 4:, this website offered visitors two pathways: the first one was clearly targeted at consumers, and the other one targeted at health and medical professionals. By 2012, readily accessible promotions for follow-on formula brands, comprising pack images and text content about product ingredients and tips about use

									were identified.
Abrahams, et al.	2012	Cross-sectional	United States	To examine the presence of infant formula marketing on popular US social media sites, using the World Health Organization International Code of Marketing of Breast-milk Substitutes as a framework.	Parents and families with infants	Then, the most popular social media (Facebook, MySpace, Google, Twitter, YouTube, and mobile applications) were identified, followed by typing the brands' and manufacturers' names as keywords in the internal search bar of each. This process was carried out for 6 months to identify infant formula promotions and related content, including sponsored reviews and mobile applications that were created or otherwise enabled by manufacturers and distributors. Each brand's website was also visited to determine the presence of interactive social media tools (e.g., message boards, photo galleries and "Tell a friend" tool). Finally, a content analysis was conducted to evaluate promotional practices using the Code as a basis for ethical marketing.	Facebook, MySpace, Google, Twitter, YouTube, Mobile Applications	Infant, follow-up, and growth formula	10 of the 11 brands identified had some social media presence. In Facebook users were prompted to "like" and to interact in the brand's posts. Five of the 11 infant formula brands had established a YouTube presence through manufacturers or brands' own dedicated YouTube channels or through promotional content distributed by a retailer. Four of the 11 brands provided free mobile apps for use by parents. In addition, five of the 11 brands contained social media features on their own Web sites.
**Foods and beverages high in saturated fat, salt and/or free sugars (FBHFSS)**									
Kelly, et al.	2021	Cross-sectional	Australia	To monitor the extent of children's exposure to web-based media food marketing as an essential step in increasing the accountability of industry and governments to protect children.	95 adolescents (13-17 years)	Children recorded their mobile device screen for two weekdays and one weekend day each time they visited relevant web-based platforms or social media. The recording process varied depending on the operating system of the mobile device, those with Android downloaded an application called *Lollipop screen recorde*r. For the iOS operating system, the screen recording settings had to be changed from the control panel of the user's device. After each day, the participants uploaded the video files to a secure server. Promoted products were defined as allowed or not allowed using the World Health Organization European Region Nutrient Profile Model.	Instagram, Facebook, Snapchat, YouTube, Twitter	Food and beverage promotions (including food and beverage products, retailers, and services)	Children saw a median of 17.4 food promotions each hour on the internet and were exposed to a median of 168.4 food promotions per week. About 99.5 of food promotions would not be permitted to be marketed based on nutrient profiling criteria. Regarding promotions, 58.77% were peer endorsed and derived from third-party sources.
Kidd, et al.	2021	Cross-sectional	New Zealand	To test the feasibility of a browser extension to estimate the exposure of adolescents to	34 adolescents (16–18 years)	A Chrome browser extension *(AdHealth*) was used to collect advertisements seen by participants on their personal Facebook accounts. The extracted information was sent to a web server where key information retrieved	Facebook	Food and beverage advertising	Among the main findings: 4% of the advertisements were food related. About 98% of the food-related advertisements were classified as not permitted.

				(un)healthy food and beverage advertisements on Facebook and the persuasive techniques used to market these foods and beverages.		included advertisement type seen and duration of each ad sighting. The WHO-Europe Nutrient Profile Model was used to classify the healthiness of products advertised.			Regarding the type of advertisements, 33.7% featured promotional characters; and 31.9% featured premium offers. The mean exposure time to unhealthy food advertisements on the users' screen was 32 s on average.
Tatlow-Golden and Boyland	2021	Report	Philippines	Main objective: To describe the extent and nature of marketing of unhealthy items in the Philippines. A second objective: To describe the nutrient content of food marketing on the social media platforms most popular with children in the Philippines.	Children and adolescents (5-17 years)	The Integrative Competitive Intelligence research company explored the online media consumption and practices of children (aged 5 to 17 years) in two urban areas in the Philippines during the COVID 19 pandemic. The research team recruited participants via Facebook and Instagram public posts to parents and guardians in order to set-up an online video or phone call to answer a survey. Filipinos' interest in less healthy foods was also explored via their online search behaviors, both before and during the Covid-19 pandemic. To carry out the social media analysis of food marketing, the process and protocols outlined in the WHO Protocols and Templates for Monitoring of Marketing of Unhealthy Products to Children and Adolescents were followed.	Facebook and Instagram	Food and brand marketing to children and adolescents on digital media.	Facebook, YouTube, and Instagram were the most popular amongst children of all ages. About 71% of children spent 6-12 hours a day on the internet during the COVID-19 pandemic. Sixty-eight percent of the children liked ads for food and beverages that included burgers, pizza, fries, fried chicken, milk tea, doughnuts, and instant noodles.<br>During the pandemic, the most popular searched and less healthy items among internet users were flavored yogurts, dairy foods, cheese, sweet foods and various flavored beverages.<br>Of the digital marketing posts, 99% were of unhealthy foods not recommended for children.<br>Social media food marketing on food brand pages in the Philippines focuses particularly on fun, taste enjoyment, family relationships, health, and nutrition.<br>Children and/or adolescents' characters, cartoons, bright colors, graphics, enjoyable activities such as games, and parent-child activities or family bonding occasions were featured in food marketing posts.
Theodore, et al.	2021	Cross-sectional	Mexico	To identify general characteristics, the use	Children and adolescents	A multistep approach was used for the sample of food/beverage of products and brands	Facebook, Twitter, and	Defined food groups: candies	The first three groups of products with the greatest

		of persuasive techniques, and the nutritional quality of Mexican digital marketing of food and beverages/brand with the greatest number of followers and views (Facebook, Twitter, and YouTube) with specific appeal to children/adolescents.		available in Mexico with the greatest number of followers and views in the commonly used social networks (Facebook, Twitter, and YouTube). The process included, as a first step, the identification of the companies' profiles with the largest audience in social media (Facebook, Instagram, and Twitter) using the *Socialbakers* platform. The following step consisted of selecting for each social media app the top 10 products/brands from three groups: 'Soft drinks'; 'Fast Moving Consumer Food' (e.g., chips, yogurt), and 'retail food'. The third step consisted of excluding duplicates and integrating a unique list. The fourth step identified the social network accounts of the selected products. Finally, the fifth step consisted of excluding accounts with no activity in the month prior to data collection. Finally, a nutritional quality assessment according to the Pan American Health Organization (PAHO) Nutrient Profile Model criteria was carried out.	YouTube	and sweets, cookies, industrial cakes, cereal bars, sweetened cereals, cola soft drinks, sweetened juices, energy beverages ice cream, yogurt, sugary milk beverages, salty snacks, pizzas, hamburgers, sausages, and breaded products.	number of followers on Facebook were: Cola and soft drinks (13,321,274); salty snacks (11,943,187) and pizzas, hamburgers, sausages, and breaded products (8,505,930).<br>On Twitter the products with the greatest number of followers were cola and soft drinks (1,020,504); sweetened juices and energy beverages (808,345) and pizzas, hamburgers, sausages, and breaded products (581,913). Finally on YouTube: pizzas, hamburgers, sausages, and breaded products (75,068,896); cola, soft drink (58,844,364) and ice cream, yogurt, and sugary milk beverages (57,407,160), were the products with the greatest number of followers. Rt6The websites and social media accounts of the food and beverage products/brands contained different identification elements of the brands and products: Logos (100%), images (93.8%), or slogans (76%). About 4 of the 46 brands evaluated asked for parental consent, and only one blocked the access linked to age (0.8%).

Bragg, et al.	2020	Cross-sectional	United States	To identify the prevalence of social media advertising among fast food, beverage, and snack companies and examine advertising techniques they use on Instagram, Facebook, Twitter, Tumblr, and Vine	Youth	In 2016 Facebook, Instagram, Twitter, Tumblr, and Vine were selected as the social media platforms to examine in this study. 200 fast food, beverage, and snack brands with the highest advertising expenditures in the United States were identified. 15 research assistants were trained to identify the official social media accounts associated with those 200 brands. The research assistants collected information on the account's followers count, post count, and first post date for each of the 200 brands between 2007 and 2016. A final subset of 20 brands was chosen for qualitative analysis where nutritional information for all foods, beverages, and snacks was collected and the development of a codebook based on the guidelines described by Lombard and colleagues was implemented to identify marketing themes. Additionally, multilevel regression was used to account for brand level variability and assessed the associations between the use of advertising themes and the presence of interactive tools or adolescents in the posts.	Twitter, Instagram, Facebook, Tumblr, and Vine	Fast food, beverages, and snacks	Among the main findings: 74.5% of the posts included for analysis features that are unique to social media and 31.5% of posts were interactive, but just 3.1% posts featured adolescents. Posts featuring adolescents were more likely to have interactive tools than posts that featured adults. A total of 982 posts appeared to target specific age groups by portraying actors in specific age groups or cartoons that appeal to children. About 9% of social media posts featured food and snack products and 29% of posts in the sample showed a beverage, where 61.2% of those posts featured a sugary beverage.
Olstad and Lee	2020	Comment	Canada	NA. This is a commentary about the importance of including artificial intelligence to strengthen the WHO CLICK methodology to monitor marketing of unhealthy food on digital media.	Children	Methodology proposal based on the CLICK monitoring framework with the addition of an artificial intelligence system to monitor unhealthy food and brand marketing to children on digital media.	Digital media	Food and brand marketing	N/S
Coates, et al.	2019	Cross-sectional	United Kingdom	The aim of the study was to explore the extent and nature of food and beverage cues featured in YouTube videos of influencers popular with children.	Children (5-15 years)	YouTube videos uploaded by two influencers popular among children in the UK were assessed using content analysis methods adapted from similar studies. Videos uploaded over a full 12-month period (January 1 of 2017 to December 31 of 2017) were analyzed over the course of a year to ensure a representative sample of products. Food and beverage items were exclusively classified as "healthy" or "less healthy" according to the UK Nutrient Profile Model. All food or beverage cues were categorized into one of five mutually exclusive	Youtube	Products high in fat, sugar and/or salt (HFSS).	There were 3571 food and beverage cues featured at an average rate of 29.9 cues per hour. Also, there was a greater prevalence and rate of less healthy cues (49.4%) than healthy (34.5%) or miscellaneous (16.1%) cues. Cakes were the most frequently featured product (9.4%), followed by fast food (8.9%). Fruits (6.5%) and vegetables

						groups (food brand, food retail establishment brand, supermarket brand, unbranded, miscellaneous).			(5.8%) featured less frequently. About 53.6% of food and beverage cues were unbranded and 29.3% were branded. Healthy cues were slightly more likely to be unbranded than less healthy cues (42.9 vs. 41.6%). Less healthy cues were much more likely to be branded (66.2%) than healthy cues. (17.1%).
Jaichuen, et al.	2019	Cross-sectional	Thailand	To assess the marketing of food on Facebook in relation to Government regulations and the industry's self-regulatory codes in Thailand.	Children and adolescents	A quantitative descriptive survey was applied to collect the marketing contents of the most popular food brands on Facebook pages. Content was analyzed to see if they comply with the Government regulation and self-regulatory codes. A coding tool against the contents of these regulations was developed. Thirty food brand pages were recorded for 24 h, every day for the whole month of December 2017. All data were collected using a screen grab from each page of the Facebook timeline and saved as PDF files for content analysis.	Facebook	3 groups of food and beverage: confectionery, soft drinks, and retail food	The most common marketing techniques applied in Facebook were the use of pictures (632 posts), followed by branding elements (569 posts) and hashtags (438 posts). Retail food groups used pictures in 90.5% of their posts. With regard confectionery and soft drink companies applied branding elements in 90.9% and 90.8%. All of the confectionery (100%) and 99.5% of soft drink pages did not display an advertising license number. About 524 of retail food posts, 184 of soft drink posts and 44 of confectionery posts had incomplete displayed information and conditions of sweepstakes.
Potvin, et al.	2019	Cross-sectional	Canada	To compare the frequency and healthfulness of food marketing seen by children and adolescents on social media apps as well as estimate their weekly exposure.	Children (7-11 years) and adolescents (12-16 years)	Participants were asked to use their two favorite social media apps for 5 minutes each. Participants wore *Tobii 12 Pro Glasses* while using social media apps. In addition, participants completed a self-administered questionnaire examining sociodemographic characteristics, ownership of electronic devices and media use.	YouTube, Instagram, Snapchat, Facebook, Twitter, Snapchat	Food marketing in general	The most used social media app during the study by children was YouTube (95%), followed by Instagram (29%). While in adolescents, the most used app was Instagram (64%) followed by Snapchat (57%). Over 72% of participants were exposed to food marketing while using their favorite social media app. Adolescents were more likely to be exposed than children (83% vs 55%). In addition, among

									children and adolescents the most promoted food categories were fast food (27% vs 50%) and sugar-sweetened beverages (10% vs 9%). It was estimated that children and adolescents view on average 0.14 and 0.26 instances of branded content per minute on social media.
Brownbill, et al	2018	Cross-sectional	Australia	To explore how sugar-sweetened beverages are marketed to Australian young people through sugar-sweetened beverage brand Facebook pages.	Young adults (14-24 years)	A content analysis of posts made by popular SSB brand pages, based on the content analysis coding frameworks and methods by Carah and Freeman et al was undertaken. The top 20 SSB pages were ranked in June 2015. We collected all official posts made by these brands to their page timeline from 1 January 2015 to 30 June 2015. Data was captured on 17 August 2015 using Capture for NVivo 10. Descriptive data for each post was collected. Thematic analysis of posts was undertaken in accordance with the guidelines suggested by Braun and Clarke using a semantic framework of people, actions and settings.	Facebook	Sugar-sweetened beverage (soft drink, sport drinks, energy drinks)	Across all brands, posts were predominantly of photos and videos (62% and 34%, respectively). Sugar-sweetened beverages were the most frequently occurring product type within posts across pages. About 70% of the marketing techniques included one or more calls to action. Brands used a range of hashtags that create a link between their posts.<br>Three interwoven themes in content posted by the SSB brands we analyzed: 'sporting prowess', 'masculinity' and 'the outdoors'.
Potvin, et al.	2018	Cross-sectional	Canada	To document the frequency and healthfulness of pop-up and banner food advertisements displayed on third-party websites preferred by adolescents in Canada.	Adolescents (12-17 years)	After a period of four months using comScore's Ad Metrix Key Measures Report, the 10 most popular websites with advertising targeting adolescents (12-17 years old), including ads from out-of-country-based food companies, were determined. The most popular websites were defined as those with a minimum of 50,000 adolescent visitors. Subsequently, by generating comScore's Ad Metrix Advertiser Report, the frequency of food ads on each selected website was identified. The nutritional analysis of the products found was based on the Nutrient Profile Model of PAHO.	Websites	Six food-related advertiser categories (i.e., food and grocery, frozen food, alcoholic beverages, restaurants, dairy, and beverages).	The five most frequently advertised food categories were cakes, cookies, and ice cream (32.5%), cold cereal (20.5%), restaurants 18.0%, 97.3% of which were for fast food<br>Most ads (93.3%) were categorized as excessive in either fat, sodium, or free sugars according to the PAHO Nutrient Profile Model.
Vandevijvere, et al.	2018	Cross-sectional	New Zealand	To analyze the extent, nature and potential impact of marketing by food and beverage brands popular in New	Adolescents (13 -18 years)	Identification of most popular food and beverage brands on Facebook and YouTube through *Socialbakers*. Numbers of page 'likes' on Facebook and numbers of channel subscribers on YouTube were used as a measure	Facebook and YouTube	Food and beverage brands on Facebook and YouTube	Among the main findings: pages with the highest potential reach were Chupa Chups and McDonald's. Coca Cola was the most popular Facebook page

				Zealand on Facebook and YouTube.		of popularity. 45 Facebook pages and 15 YouTube channels were selected to analyze two years of YouTube videos and two months of Facebook posts for nutritional quality and use of activities, promotional strategies, and premium offer.			and had the highest potential reach. About 99% of the Facebook posts of food and beverage products marketed were classified as being for occasional consumption only. Activities for consumers were used in 36% of Facebook posts. Promotional strategies were used in 41% of posts and the most frequently used strategy was having a famous sports person/team in the post or premium offers. Regarding YouTube, activities for consumers were used in 33% of videos with arts and crafts being the most common. Promotional strategies were used in 61% of videos and premium offers were used in 24% of videos.
WHO Regional Office for Europe	2018	Report	Europe	To elucidate the rapidly changing digital marketing ecosystem within which action to protect children's online experience must be taken. It then sets out two practical actions that can feasibly be undertaken: 1) the CLICK monitoring framework and 2) Proposed Policy Prerequisites.	Children	This report proposed the five-step CLICK tool to monitor the extent to which children are exposed to marketing of unhealthy products online. The five steps of the tool are: Comprehend the digital ecosystem (C); Landscape of campaigns (L); Investigate exposure (I); Capture on-screen (C), and Knowledge sharing (K).	N/S	Food and brand marketing to children on digital media.	N/S
Vandevijvere, et al.	2017	Cross-sectional	New Zealand	To assess the extent and nature of unhealthy food marketing to New Zealand children and adolescents through the internet.	Children and adolescents	The most popular websites among New Zealand children and adolescents aged 6–17 years for January 2014 were selected. Two coding tools, one for the popular, non-food websites, and one for the food brand websites, were adapted from an Australian study and used to measure the extent and nature of unhealthy food promotion. Marketing techniques and related features used on food brand websites were recorded. The	Television, sports, magazines, and Facebook	Food and brand marketing to children on digital media.	The use of promotional characters (67% vs 24%,) and tie-ins (58% vs. 44%) was more concentrated on websites targeting children and adolescents, than websites targeting the general population. Websites targeting children and adolescents offered advergames

						identification of the websites was through social media analytics (*AC Nielsen*). Products were classified according to the New Zealand Ministry of Health Food and Beverage Classification.			(25% vs 7%) and giveaways (29% vs 11%) more frequently than websites targeting the general population. Viral marketing was the most common feature and more frequent on websites targeting children and adolescents (79% vs 57%) than on websites targeting the general population.
Tatlow-Golden, et al.	2016	Research report	Ireland	To make essential first steps in identifying the digital food and drink marketing appealing to, or directed at, children and young people in Ireland.	Children and young people	Identification of 83 of Ireland's top food and drink retail brands from a listing of Ireland's 100 top retail grocery brands. Then, Google searches were conducted to locate and explore brand sites with domain addresses. For the identification of marketing techniques in Facebook, the Create Adverts feature was used to identify those brands' interests. Each brand Page was searched while logged into Facebook. Each Brand Page Timelines were analyzed in June/ July 2015. To code the Facebook brand posts, a sequence of six steps was involved to identify Facebook brand posts' themes and analyze their frequency. Parents of 13–14-year-olds were selected from a large online panel to reflect a range of demographics in Ireland and 33 took part in an online interactive interview.	Facebook and company websites	Unhealthy foods (food and non-alcoholic drinks high in fat, sugar, and salt, or HFSS)	One in five websites, almost all for items high in fat, salt, or sugar (HFSS) had content directed at or appealing to older children or teens, focusing on teen activities, entertainment and sporting celebrities. On Facebook, all the food/drink brand pages with the greatest reach among users aged 13-14 in Ireland are for brands that feature HFSS products. Only a quarter of parents were aware of food advertising online and consider many of them misleading, immoral, dishonest, and exploitative.
Freeman, et al	2014	Cross-sectional	Australia	To assess the amount, reach, and nature of energy-dense, nutrient-poor food and beverage marketing on Facebook.	Children and adolescents	The final sample of the ranked top 250 Facebook pages consisted of 27 food and beverage brand pages. For data collection the Facebook page names, the total number of Australian Facebook likes the page had received and the page's rank was recorded. Content included in the analysis was taken from all posts made by the page to its Facebook timeline from each page's launch date through February 19, 2013. A content analysis coding tool was developed to categorize the marketing techniques used by the brands. Information was collected over a 1-month period between January 19 and February 19, 2013 to estimate the reach of the marketing messages posted.	Facebook	Energy dense, nutrient-poor food	In terms of age groups, pages were most liked by those aged 18 to 24 years. Five pages (Maltesers Australia, Cold Rock Ice Creamery, Slurpee Australia, Subway Australia, Coca-Cola Australia) were most popular among those aged 13 to 17 years. Among teens between 13 to 24 years, the most common like pages were: Domino's Pizza Australia, Pringles, McDonald's Australia, and Cadbury Eyebrows. Brands' pages included photographs, branding elements, marketing techniques with celebrities, licensed characters and

								sportspeople to promote their products. More than 70% of pages included videos, quizzes or polls, links, and apps as part of their social media marketing efforts. Links to social media pages (Facebook, competitions, giveaways, or prizes) were a frequent marketing technique.
Kelly, et al.	2013	Narrative review	Australia	To identify approaches to monitoring food promotions via dominant media platforms responses by a review of studies measuring the nature and extent of exposure to food promotions	Children	Methodology proposal based on a multistep process, according to the type of media (social media, television, among others).	Television and social media	Food and non-alcoholic beverage	The 'minimal' approach involves the measurement of children's exposure to promotions. The focus should be on younger children (less than 12 years) as they are less able to distinguish between commercial and noncommercial content. The 'expanded' approach seeks to assess younger and older children's exposure to promotions. In the 'optimal' approach, measures of both extent of exposure and power of promotions across all dominant media should be collected. In all approaches, findings should be compared to existing national policies on food promotion to children and compared to international benchmarks. Analyses of the content of the promotions can be conducted quantitatively, including measuring the presence of promotional characters, premiums, or visual and audio elements and/or qualitatively, by assessing promotions' themes and emotional appeals.

BMS: Breast-milk substitutes; FBHFSS: Foods and beverages high in saturated fat, salt and/or free sugars; WHO: World Health Organization; PAHO: Pan American Health Organization; N/S: Not specified.
